# Possible Benefits of *Faecalibacterium prausnitzii* for Obesity-Associated Gut Disorders

**DOI:** 10.3389/fphar.2021.740636

**Published:** 2021-12-02

**Authors:** Tatiani Uceli Maioli, Esther Borras-Nogues, Licia Torres, Sara Candida Barbosa, Vinicius Dantas Martins, Philippe Langella, Vasco Ariston Azevedo, Jean-Marc Chatel

**Affiliations:** ^1^ Departamento de Nutrição, Escola de Enfermagem, Universidade Federal de Minas Gerais, Belo Horizonte, Brazil; ^2^ Université Paris Saclay, INRAE, AgroParisTech, Micalis, Jouy-en-Josas, France; ^3^ Programa de Pós-Graduação em Bioquímica e Imunologia, Instituto de Ciências Biológicas, Universidade Federal de Minas Gerais, Belo Horizonte, Brazil; ^4^ Departamento de Genética, Ecologia e Evolução, Instituto de Ciências Biológicas, Universidade Federal de Minas Gerais, Belo Horizonte, Brazil

**Keywords:** diabetes, gut permeability, obesity, probiotics, *Faecalibacterium prausnitzii*

## Abstract

Metabolic disorders are an increasing concern in the industrialized world. Current research has shown a direct link between the composition of the gut microbiota and the pathogenesis of obesity and diabetes. In only a few weeks, an obesity-inducing diet can lead to increased gut permeability and microbial dysbiosis, which contributes to chronic inflammation in the gut and adipose tissues, and to the development of insulin resistance. In this review, we examine the interplay between gut inflammation, insulin resistance, and the gut microbiota, and discuss how some probiotic species can be used to modulate gut homeostasis. We focus primarily on *Faecalibacterium prausnitzii*, a highly abundant butyrate-producing bacterium that has been proposed both as a biomarker for the development of different gut pathologies and as a potential treatment due to its production of anti-inflammatory metabolites.

## Introduction

Obesity has increased worldwide (Di Cesare et al., 2016) and is a public health concern across the globe. This disease is associated health outcomes such as diabetes, hypertension, and cancer (Arroyo-Johnson and Mincey, 2016). Currently, it is estimated that 30% of the global population is overweight, and this number continues to increase (Talukdar et al., 2020). The most prevalent consequence of obesity is insulin resistance and the development of type 2 diabetes (T2D). This condition is driven by inflammation that begins in adipose tissue. The hyperglycemia associated with obesity is linked with gut inflammation, increased permeability to bacterial products, and changes in microbiota composition that promote the cycle of inflammation associated with the obese state (Fallucca et al., 2014). Such pathogenic modifications of the community structure of gut microbiota are referred to as dysbiosis; one of the most well-known examples in the context of obesity is an increase in the ratio of Firmicutes to Bacteriodetes in both mice and humans, which produces a pro-inflammatory profile characteristic of the obese microbiota (Kim et al., 2012; Verdam et al., 2013).

Diet has a direct influence on intestinal inflammation, which is related to weight gain and microbiota changes. A few weeks of a high-fat, high-sugar diet is enough to induce certain gut disorders, including increased gut permeability and dysbiosis (Cani et al., 2001b). After this it is possible to detect free lipopolysaccharides (LPS) in the blood, which triggers the activation of the innate immune system and inflammatory conditions (Guerville et al., 2017).

The development of hyperglycemia has been linked with a variety of alterations in the gut mucosa in addition to dysbiosis. These include increased gut permeability, altered expression of tight junction molecules, activation of innate immune molecules, and an increase in activated adaptative immune cells. All those changes characterize a “leaky gut,” the terminology used to collectively describe those gut alterations (De Kort et al., 2011). Together, these conditions can perturb gut homeostasis and increase the probability of developing an inflammatory disease such as Crohn’s disease.

Currently, the best diet-based strategy for treating these conditions is a shift from a Western-style diet to a balanced, plant-based diet. However, the implementation of such changes takes time and requires a very high level of nutritional education. Because of this, the success rates of weight loss maintenance due to lifestyle changes are typically low (Lewis and Abreu, 2017). In response, alternative treatments are being developed that aim to ameliorate the negative gut effects. One group of treatments that has demonstrated promise are probiotics, which in certain cases have been shown to decrease both gut permeability and glycemia (Barengolts, 2016; Xu et al., 2019).

*Faecalibacterium prausnitzii* (F. prau) is a probiotic isolated from the human microbiota, where it is a dominant species in healthy adults. Its decline is associated with the development of chronic inflammation, as observed in cases of obesity. Multiple studies have demonstrated the anti-inflammatory properties of this bacterium (Feng et al., 2014; Andoh et al., 2016; Ganesan et al., 2018; Wrzosek et al., 2018), which are thought to be associated with its ability to produce butyrate. Butyrate activates the G-protein receptor (GPR) and thus facilitates downstream control of gut alterations during obesity and diabetes (Brown et al., 2003). Therefore, the goal of this review is to discuss the current state of knowledge regarding *F. prau*, particularly with respect to its potential as probiotic derived from human gut for use in alleviating the gut inflammation developed during obesity and hyperglycemia.

## Obesity and Hyperglycemia-Associated Gut Alterations

Obesity is a highly complex, multifaceted disease associated with numerous metabolic dysfunctions. These include type 2 diabetes mellitus (T2DM), dyslipidemia, cardiovascular dysfunction, and chronic inflammatory diseases (Winer et al., 2016). These conditions are linked with changes in adipose tissue (AT), a complex endocrine organ that plays an important role in energy homeostasis due to its rapid and dynamic responses to changes in nutrient availability (Sun et al., 2011). Adipose tissue is composed of adipocytes, macrophages, lymphocytes, fibroblasts, cell progenitors, and endothelial cells, and is responsible for the secretion of molecules such as leptin, adiponectin, cytokines, and the vascular regulators angiotensin II and plasminogen activator inhibitor (PAI-1) (Andersen et al., 2016).

In conditions of obesity, AT can become severely dysfunctional, with changes ranging from an increase in size to impaired function and atypical distribution in the body. This results in a suite of physiological alterations, including modifications to the extracellular matrix, vascularization, levels of oxidative stress, the profile of secreted adipokines, and the inflammatory state of infiltrated immune cells (Jo et al., 2009). Due to the increase in free fatty acids (FFA), signaling pathways such as IKK*β* and NF-*κ*B are activated, along with those linked with Toll-like receptors (TLRs) (Crawford et al., 2009; Baker et al., 2011), which influence the inflammatory state. Obese patients are typically characterized by an increase in LPS that is accompanied by higher levels of TLR4 and CD14 expression, which all contribute to the proliferation of pro-inflammatory mechanisms (Winer et al., 2016).

The activation of TLRs is important in obesity, especially TLR4 (Winer et al., 2016), as these signaling pathways regulate the phosphorylation of proteins and lead to an increase in the production of molecules such as TNF-α, IL-6, leptin, resistin, and chemokines like type 2 CC chemokine receptor (CCR2), which is related to monocyte migration. TNF-α promotes the activation of the NF-kB pathway, stimulation of the cell death signaling pathway, inhibition of the expression of the glucose transporter GLUT-4, and an increase in FFA levels and consequent reduction in insulin sensitivity (Gomez-Hernandez et al., 2016).

Compared to their lean counterparts, mice that are fed a high-fat diet present a higher number of TCD4^+^ and TCD8^+^ cells and higher levels of IFN-*γ* and TNF-α, mainly in adipose tissue (Lumeng et al., 2007). The infiltration of TCD8^+^ cells in adipose tissue is followed by the accumulation of CX3CR1^int^ macrophages, which migrate towards AT in response to greater amounts of FFAs, glucose, and apoptosis, thus increasing inflammation (Rocha et al., 2014). Adipose tissue macrophages (ATMs) infiltrate adipose tissue in a CCR2-dependent manner and inhibit the insulin signal in insulin-sensitive tissues, including liver, adipose tissue, and muscle (Hotamisligil, 2017; Kawano et al., 2016). Taken together, these conditions contribute to the production of pro-inflammatory cytokines and the release of monocyte 1 and 3 chemotactic protein (MCP-1 and MCP-3), which creates a cycle of continuous cell recruitment and constant inflammation in AT (Nishimura et al., 2009).

The inflammatory state in visceral adipose tissue, along with an excess of metabolites in the circulation, is a major driver of obesity-related insulin resistance (IR). IR is characterized by impaired phosphorylation of the insulin receptor in cells that depend on insulin. This results in increased serine phosphorylation of insulin receptor substrate 1 and 2 (IRS-1 and IRS-2) and activation of the SOCS (suppressor of cytokine signaling) protein, which reduces the insulin receptor’s ability to transmit signals downstream in the insulin pathway and to capture glucose in cells (Biddinger and Kahn, 2006).

When the full extent of metabolic dysfunction is considered, the physiological impact of obesity is wide-ranging, with numerous effects on the architecture and functionality of primary and secondary immune system organs, including bone marrow, thymus, and lymph nodes (Yang et al., 2009). The accumulation of lipids reduces hematopoiesis in bone marrow and thymopoiesis in the thymus, which is added to a restricted diversity of T cell receptor repertoires in the thymus (Yang et al., 2009). In the peripheral immune response, there is a reduction in the migration of antigen-presenting cells to peripheral lymph nodes and a consequent reduction in the differentiation of naïve T cells into effective cells. These systemic effects can also translate into disturbances to the homeostasis of gut mucosa, which together with the mucosal-associated lymphoid tissue harbor the majority of immune cells found in the body.

The intestinal mucosa is the largest surface of the human body that is in contact with the external environment (Rezende and Weiner, 2017). The intestinal immune system is thus faced with an immense challenge: tolerating the vast amount of antigens that originate from the diet and the commensal microbiota while, at the same time, protecting against intestinal pathogens and toxins (Faria et al., 2017). Alterations in the intestinal barrier and dysbiosis in the gut of obese individuals may not only favor the development of diseases, but may also compromise immune tolerance to dietary antigens and the microbiota (Spiekermann and Walker, 2001; Faria and Weiner, 2005).

In the literature, there is an abundance of evidence on the many ways obesity and insulin resistance can affect immune responses and gut physiology. Obesity enhances jejunal inflammation and increases the density of macrophage populations, CD3+ T cells, intraepithelial lymphocytes and mature dendritic cells. It also increases the expression of pro-inflammatory cytokines (IFN*γ*, IL1β, TNFα), chemokines, and co-stimulatory factors in cells from the lamina propria and epithelial compartment. The increase in T-cell populations in the intestinal mucosa of obese subjects has also been associated with an impaired insulin response compared with lean subjects (Monteiro-Sepulveda et al., 2015). In humans with obesity and insulin resistance, there is a stronger inflammatory profile in the duodenum compared to non-obese patients, with more inflammatory cytokines and M1 macrophages (Ho-Plagaro et al., 2019). In addition, obesity and excess weight have been associated with increased gut permeability—demonstrated by increased serum concentrations of zonulin—along with microbiota modifications (Mörkl et al., 2018). Increased gut permeability was also reported in individuals with high levels of fasting glucose, as well as higher levels of pro-oxidative markers in the blood compared with healthier subjects (Carnevale et al., 2017). Furthermore, intestinal permeability was found to be sharply increased by a high-fat diet (HFD), probably mediated by a reduction in the expression of ZO-1 and occludin, which then favors the translocation of LPS through the intestinal wall (Cani et al., 2008b).

Hyperglycemia associated with HFD increases levels of IFN-*γ*– and IL-17–producing inflammatory cells in the intestine and intestinal permeability, and decreases the abundance of regulatory cells (Luck et al., 2015). Even in the absence of excess weight, hyperglycemia has been associated with increased permeability and alterations in the mucosal immune system. For example, in *db/db* mice with controlled food intake or in mice treated with streptozotocin, high levels of blood glucose were associated with high intestinal permeability and increased expression of PRP (pathogen recognition patterns) in lymphoid organs (Thaiss et al., 2018). In other words, hyperglycemia that is secondary to obesity, IR, or even T1D can further compromise intestinal health. Alterations in glucose metabolism can also decrease oral tolerance of antigens from the diet (Miranda et al., 2019) and increase the severity of food allergies (data not published) in mice.

**FIGURE 1:**
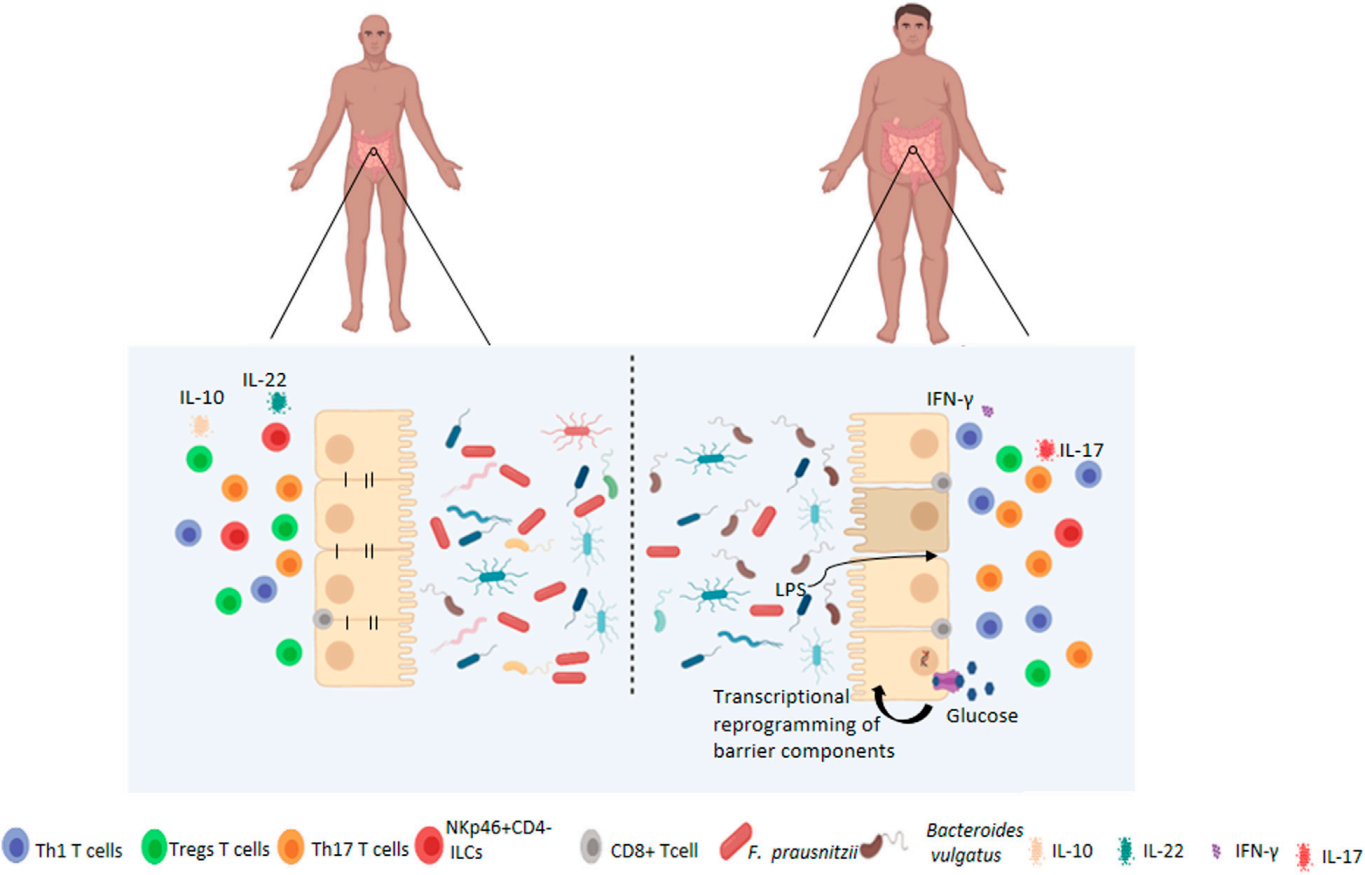
Changes to the intestinal barrier functions associated with obesity. Under normal circumstances, the gut microbiota is highly diversified which contributes to the maintenance of intestinal permeability, tolerance to dietary antigens and immunoregulation. Obese patients have dysbiosis of the gut microbiota, characterised by decreased diversity, and altered barrier functions such as increased permeability and activation of pro-inflammatory pathways by immune cells at local and systemic levels. Obesity-associated hyperglycemia may also drive barrier permeability through transcriptional reprogramming resulting in decreased expression of adherens and tight juction proteins.

One of the main consequences of the development of inflammatory alterations and the breakdown of gut epithelial integrity is the leakage of bacterial products, including endotoxins such as LPS, across the intestinal epithelium. This then results in dysfunction of immune organs, inadequate distribution of leukocyte populations, and changes in lymphocyte activity, which can all affect the immune response against pathogens, regulation of the immune response to dietary antigens, and the intestinal microbiota (Figure 1).

## Changes in Gut Microbiota Related to Obesity and Hyperglycemia

Composition of the microbiota is modulated by the availability of dietary nutrients that provide a wide variety of essential metabolites for the maintenance of intestinal architecture and integrity, while simultaneously acting on the modulation of immunity. In healthy conditions, Bacteroidetes and Firmicutes are the most abundant phyla of the microbial gut community. Although the structure of these communities can vary, some general patterns have been noted. For example, studies have determined that some of the most abundant intestinal bacterial species in healthy individuals tend to include members of the *Dorea/Eubacterium/Ruminococcus* groups as well as Bifidobacteria, Proteobacteria, and streptococci/lactobacilli (Eckburg et al., 2005; Qin et al., 2010).

Diet is the key determinant of microbiota composition; it modulates the abundance of various species and, consequently, their individual or collective functions. In humanized gnotobiotic mice, a shift from a low-fat, plant polysaccharide–rich diet to a high-fat and high-sugar diet had detectable effects on microbial community structure and metabolic pathways after only a single day. More specifically, the increase in body fat percentage in mice fed a high-fat diet was positively associated with the abundance of species in the genera *Lactococcus* and *Allobaculum* but was negatively associated with *Akkermansia* (Kolodziejczyk et al., 2019).

The alterations in the microbiota linked with HFD-induced obesity also have effects on gut permeability to bacteria and bacterial products. The Burcelin group (2008, 2011) described that after 1 week of HFD consumption, changes could be seen in the intestinal mucosa and microbiota such as co-localization of bacteria with dendritic cells (DCs) both in the mucosa and in the mesenteric lymph nodes. After 4 weeks of HFD consumption, clear increases in intestinal permeability have been noted, with a concomitant decrease in zonulin expression in the intestine. These changes were also dependent on changes in the microbiota (Cani et al., 2008b; Amar et al., 2011). These alterations in the intestine permit increased bacterial translocation, which involves intestinal phagocytes and requires the recognition of pathogen-associated molecular patterns (PAMPs) by standard recognition receptors (PRRs), such as TLRs and nod-like receptors, to activate an innate immune response (Kim et al., 2012; Luck et al., 2015). Under pathological conditions. However, this molecular signaling process can favor further infiltration and cell accumulation in adipose tissue, triggering local inflammation.

The relationship between dysbiosis and gut permeability also plays a role in the development of insulin resistance. Specifically, alterations in the abundance of intestinal bacteria caused by chronically elevated glucose levels and obesity are known to result in dysfunction in the intestinal barrier (Guerville et al., 2017). As a result, high concentrations of gram-negative bacterial cell-wall products, such as LPS, can cross the intestinal barrier to reach other organs and tissues, and induce chronic inflammation (Cani et al., 2009). This can initiate a series of inflammatory mechanisms, setting in motion one of the main processes that leads to insulin resistance and ultimately T2D (Cani et al., 2007; Ganesan et al., 2018). This can be further exacerbated by the fact that the production of pro-inflammatory cytokines interferes with insulin secretion and the expression of insulin mRNA in human beta islet cells. Changes in the composition of the microbiota can thus play a multi-faceted role in intestinal barrier dysfunction and, consequently, in metabolic disorders.

Certain groups of bacteria have been linked with various conditions in the gut. As mentioned earlier, an increased ratio of Firmicutes to Bacteroidetes in obese individuals has been related to inflammatory diseases (Turnbaugh et al., 2006), while conversely, the ratio of *Bacteroides* to *Prevotella* is lower in obese subjects than their lean counterparts. *F. prau* has been negatively associated with insulin resistance (Furet et al., 2010), while potential proinflammatory bacteria such as *Ruminococcus gnavus* or *Bacteroides* may dominate the microbiota of obese patients. In general, a reduction of butyrate-producing bacteria in obese subjects has been associated with an increase in mucus degradation (Cotillard et al., 2013). Obese women with high serum levels of zonulin, which is correlated with higher gut permeability, showed decreased abundance of Ruminococcaceae and *Faecalibacteri* both could weaken the gut barrier and lead to systemic inflammatory responses (Mörkl et al., 2018).

A study in mice reported that, after 1 week of HFD, and the subsequent early onset of diabetes, gram-negative bacteria start to adhere in the DCs of the gut mucosa; this increased bacterial translocation and triggered inflammation (Amar et al., 2011). Interestingly, mice that are deficient in TLR5, which recognizes bacterial flagellin, develop obesity and features of metabolic syndrome even in the absence of a high-fat diet, solely as a function of microbiota changes (Vijay-Kumar et al., 2010). Finally, some bacterial species have been identified as particular promoters of insulin resistance, for example, *Prevotella copri* and *Bacteroides vulgatus*, which were noted to be the main species driving the association between the biosynthesis of branched-chain amino acids and insulin resistance in humans (Pedersen et al., 2016).

One important function of the microbiota is the metabolism of polysaccharides, through which these bacteria produce a wide variety of metabolites, such as short-chain fatty acids (SCFAs), that are essential for the microbial population and for the maintenance of intestinal homeostasis (Sun et al., 2017). The SCFAs acetate, butyrate, and propionate are the main products of fermentation in the intestine, and are of particular interest due to their ability to activate local G-protein-coupled receptors (GPCR) in epithelial cells, especially GPR41 and GPR43 (Miyamoto et al., 2016). This process has feedback effects on the physiology of the intestinal microbiome and mediates chronic inflammation, affecting both glucose homeostasis and insulin sensitivity (Ang and Ding, 2016; Lambertz et al., 2017). Butyrate-producing bacteria such as *F. prau* can reduce bacterial translocation and stimulate mucin secretion, which acts to maintain the integrity of the intestine (Ganesan et al., 2018).

Interactions between the intestinal microbiota and host cells require finely tuned control by the immune system, as specialized cells are needed to recognize bacterial fragments and induce inflammation when appropriate. A significant consequence of the microbial changes associated with obesity is activation of the innate immune system, which often results in chronic inflammation (Cani et al., 2007; Cani et al.,2008a). In animal models, increased levels of LPS in the bloodstream can directly damage pancreatic *β* cells and increase insulitis by triggering the innate immune response; this is a crucial factor in the pathogenesis of insulin resistance (Ganesan et al., 2018). Similarly, Amar et al. (2011) reported that excess LPS fragments in the blood of diabetic mice induced adipose tissue inflammation.

Interestingly, when HFD experiments are performed in animals lacking the microbial pattern recognition receptors Nod1 or CD14, diet no longer has strong effects on bacterial permeability and translocation (Amar et al., 2011). Likewise, TLR4-knockout mice have a completely different microbiota and display elevated blood LPS levels and diabetes development compared to WT controls. These alterations in gut morphology and microbiota composition are also correlated with lower levels of circulating SCFAs, which suggests an interaction between intestinal functions, microbiota composition, and the development of diabetes (Simon et al., 2020).

Taken together, the changes to the structure and function of the gut microbiota that occur in cases of obesity and hyperglycemia have the potential to severely exacerbate mucosal inflammation and gut permeability. For this reason, the use of bacterial probiotics—especially those known to produce high levels of SCFAs—could represent an interesting approach to treat the damage associated with intestinal metabolic syndrome.

## Probiotics as an Alternative Treatment for Obesity and Hyperglycemia

Modulation of the microbiota has become a regular and effective approach in the treatment and prevention of mucositis, colorectal cancer, neurological diseases, and several other disorders (Sanders et al., 2019). There are several ways to modify the microbiota, including diet alteration; the administration of probiotics, prebiotics, and postbiotics; and fecal microbiota transplantation. The underlying goal of all of these methods is the same: to modify the bacterial composition in the gut in order to provide benefits to the host. As the links between obesity, hyperglycemia, and alterations in the microbiota have become clearer, studies have attempted to shed light on the exact mechanisms by which diet is able to affect the microbiome. It is possible to envision that microbiota modulation could serve as a non-invasive means of treating metabolic conditions, especially obesity, and thus represent an attractive alternative to common approaches such as bariatric surgery.

As mentioned above, obesity-related dysbiosis induces a series of events, mainly in the intestine, that result in chronic inflammation and disruptions to intestinal homeostasis. However, until the early 2000s, there was no discussion of the possibility that the microbiota itself could contribute to weight gain (Backhed et al., 2004). The Gordon group has conducted research on this topic, and their pioneering studies proposed an interesting hypothesis—that the microbiome from obese subjects has an increased capacity to harvest energy from the diet. First, it was reported that control mice colonized with an “obese” microbiota present higher body fat compared to mice colonized with “lean” microbiota (Turnbaugh et al., 2006). Further studies with gnotobiotic germ-free C57BL/6 mice revealed that a Western diet induced a drop in microbiota diversity, with an increase in abundance of a single class of Firmicutes. In addition, when the microbiota from conventionally raised obese mice were transplanted into wild-type mice, they showed a greater increase in body fat (Turnbaugh et al., 2008). Finally, a fascinating study with adult monozygotic and dizygotic twins showed that, although twins shared a core microbiota, obese siblings had less diversity in their microbial assemblages, lower proportions of Bacteroidetes, and a higher proportion of Actinobacteria compared to their leaner twins (Turnbaugh et al., 2009). These interesting findings have led to the generation of several hypotheses, with one of the most intriguing being that microbiota modulation by itself might be an effective treatment for obesity.

The relationship between gut microbiota and energy balance (and thus the development of larger adipose tissue) is complex because it involves many factors, such as diet, gender, and culture, among others. However, recent studies have pointed to the gut microbiome as a key element in the regulation of food absorption; there is evidence that the gut microbiota can affect hormone secretion directly in the brain, in areas that are responsible for controlling appetite, fat storage, and energy expenditure (Cerdó et al., 2019). Although the science is far from settled on the topic, based on these initial results, some attempts have been made to treat obesity by modifying the host microbiota.

To date, probiotics and prebiotics have been used for the treatment of obesity in both experimental models and clinical trials. Probiotics are live microorganisms that, when ingested, can confer benefits on the host, while prebiotics are molecules or components (such as food fiber) that are capable of modifying the composition of the intestinal bacterial assemblage or stimulating the activity of one or a limited number of bacterial species in a positive way (Gibson et al., 2017). In most cases, one of the noted benefits of both prebiotics and probiotics is increased levels of SCFAs, which have regulatory effects and are important for intestinal integrity. Much research has focused on the positive effects of *Lactobacillus* and *Bifidobacterium* in obesity; both bacteria have been reported to contribute to weight loss and a reduction in insulin resistance. For instance, several studies involving different mouse or rat models of obesity have shown positive results from treatment with certain strains of *L. plantarum* (DSM 15313, Strain No. 14, and TL8), such as less weight gain, reduced adiposity, and higher insulin sensitivity (Axling et al., 2012; Ben Salah et al., 2013; Okubo et al., 2013). A recent publication reported that *L. plantarum* LMT1-48 significantly reduced weight in HFD-fed mice and its extract was capable of inhibiting the differentiation of adipocytes through downregulation of genes such as PPAR-*γ* (Choi et al., 2020). Notably, synergistic effects were observed from a probiotic combination of *L. plantarum* KY1032 and *L. curvatus* HY7601; HFD-fed mice treated with this mixture showed reduced body weight gain and fat accumulation, lower levels of insulin and cholesterol, and fewer biomarkers for inflammation (Park et al., 2013; Yoo et al., 2013). One exception was reported with HFD-fed mice treated with *L. plantarum* DSM 15313, which showed an increase in body weight despite lower levels of glucose in plasma (Andersson et al., 2010). Another study, with *L. plantarum* NCIMB8826, found no effect on body weight (Martinic et al., 2018). Overall, numerous strains—including *L. paracasei* CNCM I-4034, *L. casei* IMVB-7280, *L. paracasei* HII01, *L. casei* IBS041, *L. rhamnosus* CGMCC1.3, *L. rhamnosus* PB01 (DSM 14870), and *L. rhamnosus* LA68, among others—have demonstrated probiotic potential in studies of obesity, inducing positive effects such as reduced weight gain, less adiposity with less white adipose tissue, and reduced cholesterol levels, as reviewed by Ejathed et al. (Ejtahed et al., 2019).

Avolio and colleagues et al showed that HFD-fed hamsters treated with a probiotic mix of six species (*S. thermophilus, L. bulgaricus, L. lactis, L. plantarum, B. lactis*, and *L. reuteri*) presented a reduction in body weight and reduced levels of inflammatory factors in the blood compared to control HFD animals (Avolio et al., 2019). A study of leptin-deficient mice (ob/ob mice) showed that administration of the plant-derived lactic acid bacterium *Pediococcus pentosaceus* was sufficient to reduce adipocyte size and liver triglyceride content (Zhao et al., 2012). Similarly, HFD-fed mice treated with *L. plantarum* demonstrated reduced adipose tissue and triglyceride levels; the treatment also reduced the Firmicutes/Bacteroidetes ratio and improved the gut microbiota composition (Joung et al., 2021). Finally, although most effort to date has focused on bacteria, the most-studied yeast probiotic, *Saccharomyces boulardii,* has been reported to reduce hepatic steatosis and hepatic inflammation in db/db mice (Everard et al., 2014).

Positive results are also being reported from obese mice treated with various types of prebiotics. For example, the use of oligofructose as a prebiotic in HFD-fed obese mice resulted in higher numbers of intestinal *Bifidobacteria* and *Lactobacillus* in treated mice; this correlated with higher zonulin expression, leading to less intestinal permeability and less hepatic inflammation (Cani et al.,2008b). The addition of oligofructose in the diet was also found to promote satiety in HFD-fed mice, contributing to a reduction in both weight and adipose tissue deposits (Cani et al., 2005; Régnier et al., 2021). Another study showed that short-chain fructose-oligosaccharides induced a significant increase in the abundance of *Bifidobacteria* in obese mice, and these animals gained less weight than control HFD mice (Respondek et al., 2013). In general, the use of prebiotics as a treatment for obesity seems quite promising; this is especially true for approaches using food fiber, which seems to increase the production of satiety hormones, thus helping to control weight and increasing sensitivity to insulin (Parnell et al., 2012).

In humans, several clinical trials of probiotics and prebiotics as a treatment for obesity have reported positive results. For example, a randomized, double-blind study conducted by Osterberg and collaborators demonstrated that administration of a probiotic containing eight strains of bacteria (*Lactobacillus*, *Bifidobacterium*, and *Streptococcus*) attenuated increases in body mass index (BMI) and adipose tissue in subjects on a high-fat diet (Boutagy et al., 2015). Similarly, other studies of interventions with *Lactobacillus* spp. and *Bifidobacterium* spp. have reported positive results in obese and overweight subjects, such as less body weight and less fat storage (Minami et al., 2015; Madjd et al., 2017). Two recent systematic reviews concluded that some probiotic strains are effective in reducing BMI and hip circumference (Shirvani-Rad et al., 2021; Tomé-Castro et al., 2021).

Despite the promising results thus far, there is still much room for new approaches and improvements, especially those aimed at elucidating the mechanisms by which various probiotics and prebiotics induce reductions in weight and inflammation. In this context, the probiotic derived from human gut *F. prau* may prove particularly useful.

## *F. prau* as a New Treatment to Improve Gut Homeostasis During Obesity and IR

*F. prau* is a Gram-positive bacterium belonging to the Ruminococcaceae family, in *Clostridium* cluster IV. It is one of the most abundant species found in the gut, representing between 1 and 6% of the total fecal microbiota (Hold et al., 2003). Multiple studies have described its anti-inflammatory properties and its role in tissue damage repair and protection against colitis(Sokol et al., 2008; Martín et al., 2014; Rossi et al., 2015), which appear to be related, at least in part, to its ability to produce butyrate(Zhou et al., 2018). *F. prau* is characterized by great intra-species diversity; indeed, it has been suggested that the genomic disparities are great enough to warrant separating the group into at least two different species: *F. prau* sensu stricto and *F. moorei* sp. nov (Fitzgerald et al., 2018).

Patterns of abundance of *F. prau* and its different phylogroups have been associated with various pathologies of the gut. For example, IBD patients were found to host reduced phylotype richness of *F. prau* compared to a healthy group. Specifically, total levels of *F. prau* phylogroup I were reduced, and this pattern could be used to accurately differentiate IBD and colorectal cancer patients from healthy subjects; instead, phylogroup II was specifically reduced in Crohn’s disease (CD) patients. Interestingly, *F. prau* prevalence was found to be reduced locally in either the ileum, colon, or rectum depending on the form of the patient’s CD (Lopez-Siles et al., 2016). Taken together, this demonstrates the potential applications of *F. prau* phylotypes as biomarkers for the diagnosis and prognosis of patients.

As discussed earlier, diversity of the microbiota is lower in obese patients, and *F. prau* has been implicated in these changes in community composition. For example, analysis of a Chinese cohort revealed a reduced abundance of *Bacteroides*, *Akkermansia* (another butyrate-producing bacterium), and *F. prau* in T2D patients. Evidence of this shift was even detectable in pre-diabetic obese patients, reflecting the link between glucose intolerance and microbiota composition (Zhang et al., 2013). These results—especially with respect to *F. prau*—were corroborated in a later Iranian study, which also reported a negative correlation between *F. prau* count and BMI (Navab-Moghadam et al., 2017). In another study, though, *F. prau* was found to be enriched in patients suffering from T2D after weight loss (Hippe et al., 2016). Thus, although the exact mechanisms have yet to be determined, the current state of research supports a link between BMI, blood glucose levels, and the abundance of *F. prau*.

Another interesting finding with respect to *F. prau* was the observation of differences in gut composition on the basis of gender, especially for this bacterium and streptococci. In a population of obese Chinese patients, a positive correlation was found between *F. prau* abundance and fasting glucose levels in men but not in women (Aguirre de Cárcer et al., 2011).

Rapid improvements (on the scale of a few days) have been observed in the community structure of gut microbiota as a result of nutritional interventions. Diets rich in non-digestible carbohydrates, such as inulin-type fructans, fructooligosaccharides, polydextrose, soluble corn fiber, and raffinose, have been observed to increase the abundance of *F. prau* (Verhoog et al., 2019). Supplements can also be effective; for example, kiwifruit-based supplementation was noted to increase *F. prau* abundance in the gut as well as stool frequency in humans (Rush et al., 2002; Blatchford et al., 2017).

Alternatively, supplementation with *F. prau* itself could have potential in the treatment of obesity and its associated disorders. HFD-fed mice that were treated twice a week with *F. prau* displayed decreased hepatic inflammation, with fewer lipids accumulated in the liver, as well as a reduction in cell infiltration of adipose tissue and adipocyte size compared to controls (Munukka et al., 2017).

**FIGURE 2:**
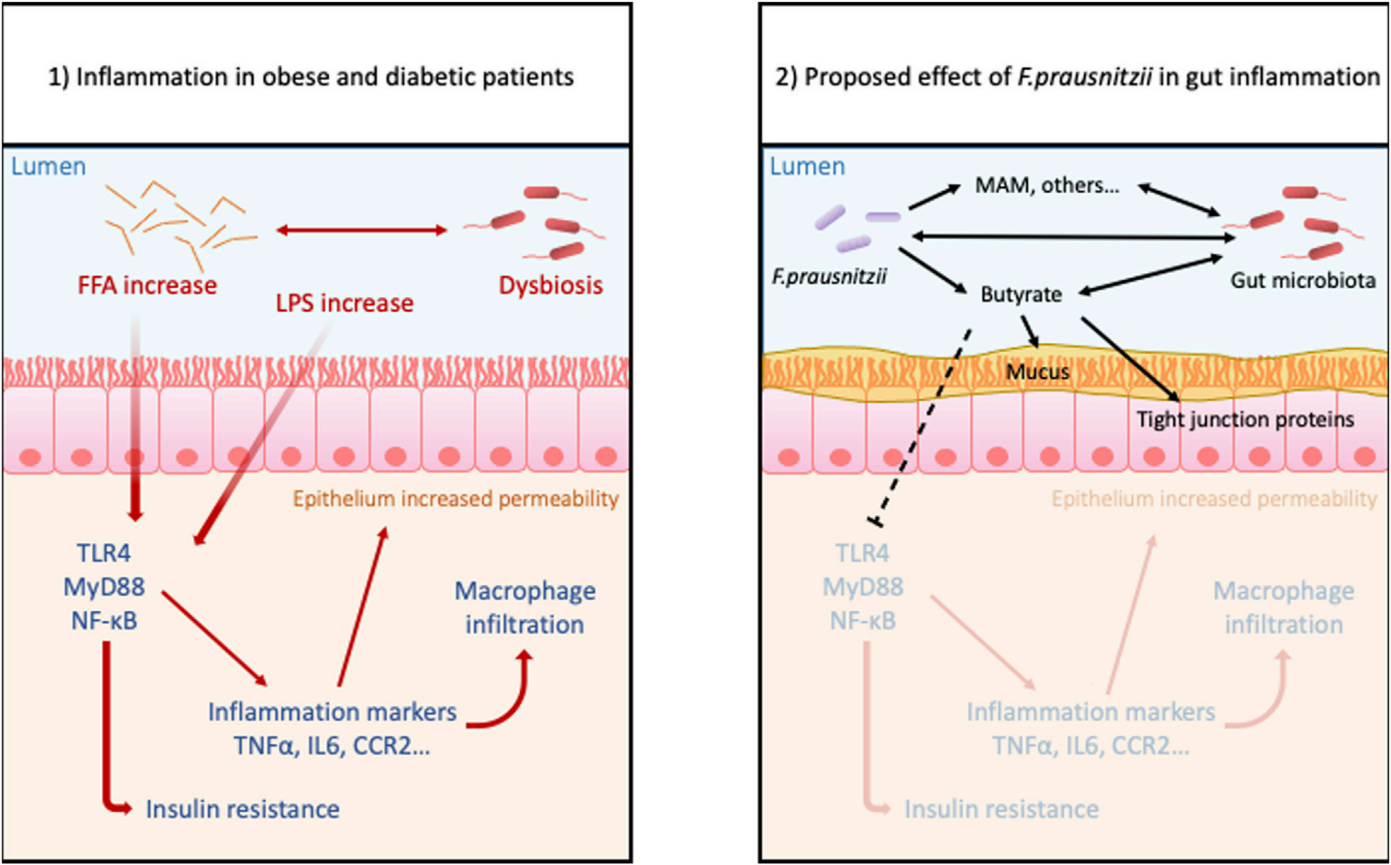
Proposed benefits effects of *Faecalibacterium prausnitzii* in gut inflammation due to obesity: Obesity and hyperglycemia cause dysbiosis, increased level of FFA and LPS, and also disrupts intestinal permeability. These conditions trigger inflammatory pathways in the lamina propria allowing more permeability to antigens and bacteria. This leads to a leaky gut and susceptibility to inflammatory disorders. The treatment with probiotic F. prau and its products can increase the butyrate concentration, stabilizes the microbiota and mucous layer and decreases the activation of inflammatory pathway.

Finally, many studies have examined the mechanisms by which *F. prau* exerts its beneficial impacts on the intestinal health of obese individuals (Figure 2). It appears that these effects may not only be a function of the abundance of this bacterium in the intestine, but also of the quantity and type of metabolites it produces, such as butyrate. Indeed, some of these metabolites have already demonstrated promise for the treatment of obesity and T2D (Ganesan et al., 2018; Verhoog et al., 2019).

## *F. prau* Action Through Butyrate Production

*F. prau* is one of the most abundant butyrate-producing bacteria in human feces (Hold et al., 2003), and this SCFA is currently the subject of intense research focused on its positive health effects. For example, butyrate can prevent HFD-induced insulin insensitivity through epigenetic regulation that increases mitochondrial beta-oxidation, thus improving glucose sensitivity and adiposity (Fernandes et al., 2014).

The butyrate-containing supernatant of *F. prau* has been found to regulate Th17/Treg differentiation through inhibition of the IL-6 and STAT3/IL-17 proinflammatory pathway, specifically by targeting histone deacetylase 1 (HDAC1) (Rivière et al., 2016; Zhou et al., 2018). Although this mechanism was demonstrated in a colitis model, this proinflammatory pathway is also involved in obesity (Hippe et al., 2016). These results are consistent with the observation that HFD-mice treated with *F. prau* have improved hepatic health and reduced inflammation in adipose tissue (Munukka et al., 2017).

The anti-inflammatory properties of butyrate, and by extension *F. prau*, have long been known to be beneficial to IBD patients(JM et al., 1989; W et al., 1992; Scheppach, 1996). A recent clinical study found that administration of encapsulated sodium butyrate altered patients’ gut microbiota by increasing the abundance of SCFA-producing bacteria in ulcerative colitis patients and butyrate-producing bacteria in Crohn’s disease patients, with the former group reporting an increased quality of life (Facchin et al., 2020). Sodium butyrate supplementation also had positive effects on HFD-fed mice by altering the composition of the gut microbiota, lowering serum LPS concentration, and reducing HFD-induced inflammation (Zhou et al., 2017).

The A2-165 strain of *F. prau* has been found to induce a distinct cytokine response, with high IL-10 secretion compared to other *F. prau* strains tested (Rossi et al., 2016). This may be the result of higher butyrate production, which is known to induce IL-10 responses in Th1 cells (Sun et al., 2018). Indeed, a recent study demonstrated that this strain’s anti-inflammatory properties in primary human colonic mucosal barrier cells are primarily due to the downregulation of TLR3 and TLR4 by butyrate (Zhang et al., 2021). It thus seems likely that differences in the cytokine profile induced by strain A2-165 compared to other strains of *F. prau* can be at least partially explained by higher butyrate production.

However, gaps remain in our understanding of *F. prau’s* butyrate metabolism and its effects on patients’ health, with some studies reporting inconsistent results. For example, an early study in southern India found significantly higher abundances of *F. prau* in obese children compared to non-obese children (Balamurugan et al., 2010). Similarly, Pinto et al. (2017) found increased levels of *F. prau* in T1D patients. They showed that the high abundance of the gluconeogenic enzyme phosphenolpyruvate carboxykinase in the gut proteome of their T1D cohort could be attributed to two strains of *F. prau* and hypothesized that this was due to low levels of glycolytic sources in the diets of T1D patients and a lack of acetate—necessary for butyrate production—from other bacterial sources such as *Bifidobacterium* spp. (Pinto et al., 2017). This hypothesis was supported by the finding that co-culture with strains of *Bifidobacterium* improved growth, gut colonization, and butyrate production of *F. prau.* Administration of the co-culture supernatant to mice decreased DSS-induced inflammation, providing further evidence that the beneficial properties of *F. prau* are dependent on its surrounding environment and interactions with other species (Ganesan et al., 2018; Kim et al., 2020).

Intriguingly, a 2016 study reported that samples from lean patients contained the highest count of *F. prau* genes compared to obese and T2D patients, but the lowest content of the *F. prau*–associated butyryl-CoA:acetate CoA-transferase (BUT) gene. Instead, T2D patients demonstrated the highest BUT content. This was interpreted as evidence that different phylotypes of *F. prau* produce different levels of butyrate *in vivo* and that their abundance differs from healthy to unhealthy patients (Hippe et al., 2016). It seems paradoxical that higher butyrate production by *F. prau* would be associated with obesity and T2D. The authors hypothesized that, while certain levels of butyrate production can be protective in obese patients, greatly increased production can lead to inflammation in the gut and the development of T2D. The dose-dependent effect of butyrate on epithelium permeability has long been supported by work using transepithelial electrical resistance measurements; experiments on Caco-2 cells showed protective effects at 2 mM and detrimental effects at 8 mM of butyrate (Peng et al., 2007). Similarly, a Belgian study on primary cell monolayers from ulcerative colitis patients found that although butyrate had protective effects in control non-inflamed tissue, it actually worsened inflammation when administered together with TNF-alpha and IFN-gamma, resulting in a dramatic increase in IL-8 production (Vancamelbeke et al., 2019).

*F. prau* has been associated with the production of several other metabolites, including shikimic and salicylic acids, known for their antimicrobial activity, and *α*-ketoglutaric acid, which is involved in ammonia recycling and cell proliferation and differentiation, and known to be depleted in patients with gut dysbiosis (Miquel et al., 2015). Investigation of these other metabolites has become all the more relevant given a report that the ability of 13 different strains to decrease IL-8 levels induced by TNF-α stimulation in HT-29 cells was not correlated either to growth ratio or butyrate production (Martín et al., 2017).

Quévrain et al. (2016) identified a family of peptides from *F. prau* that were all derived from the same protein, the microbial anti-inflammatory molecule (MAM). They demonstrated that the anti-inflammatory properties of MAM arose through inactivation of the NF-*κ*B pathway. Later experiments in models of DNBS- and DSS-induced colitis validated the effects of MAM on NF-*κ*B *in vivo* and demonstrated its ability to inhibit the Th1 and Th17 immune responses (Breyner et al., 2017).

A more recent investigation of MAM analyzed its effects in db/db mice, which do not express leptin receptors and which demonstrated a depressed abundance of *F. prau* in the gut. When these mice were supplemented with MAM produced by *E. coli*, this protein was found to interact with ZO-1 and other tight junction proteins. Furthermore, the transfection of MAM into a cell line was able to increase ZO-1 expression and restore epithelial barrier function (Xu et al., 2019). These results suggest that MAM could have potential as an alternative treatment for pathologies involving disturbances of the gut epithelium and gut permeability.

It has thus become evident that butyrate is far from the only metabolite implicated in the immunomodulatory properties of *F. prau*. The use of this bacterium as a preventive or complementary treatment for obesity- and T2D-related inflammation of the gut could be promising. However, we first need a better understanding of the optimal dosage of bacterial units and the effects of butyrate production on host health, as well as how *F. prau* and butyrate metabolism are affected by other gut bacteria.

## Final Consideration

Obesity is a metabolic disease caused by several factors—genetic, environmental, hormonal, and behavioral—but mainly by excess energy intake. It predisposes patients to other diseases such as hypertension and type 2 diabetes, and it is also associated with disorders of intestinal homeostasis, such as increased permeability and dysbiosis. Non-surgical treatments for obesity include changes in lifestyle, diet, and medications, all of which tend to have low adherence by the patient and are rarely effective for weight control or even for shaping intestinal health. As a major factor associated with obesity and gut health, intestinal dysbiosis may be the key to the effectiveness of weight management and the control of its associated comorbidities. For this reason, several probiotics have been tested successfully for the control of obesity, and there is intense interest in the discovery of new potential treatments.

*F. prau* is well known as an abundant bacterium in the natural human microbiota whose abundance is reduced in obese individuals. In addition to being a high producer of butyrate, it has anti-inflammatory effects that contribute to intestinal homeostasis. Therefore, the use of *F. prau* or its derivative products may represent a good alternative for the treatment of intestinal disorders linked with obesity and its comorbidities. However, studies on dose, forms of administration, and mechanisms of action are still necessary in order to improve our understanding of the most appropriate use of this bacterium.
